# Contribution of Environment and Genetics to Pancreatic Cancer Susceptibility

**DOI:** 10.1371/journal.pone.0090052

**Published:** 2014-03-20

**Authors:** Barbara A. Hocevar, Lisa M. Kamendulis, Xinzhu Pu, Susan M. Perkins, Zheng-Yu Wang, Erica L. Johnston, John M. DeWitt, Lang Li, Patrick J. Loehrer, James E. Klaunig, E. Gabriela Chiorean

**Affiliations:** 1 Department of Environmental Health, Indiana University, Bloomington, Indiana, United States of America; 2 Indiana University School of Medicine, Indianapolis, Indiana, United States of America; 3 Indiana University Melvin and Bren Simon Cancer Center, Indianapolis, Indiana, United States of America; 4 University of Washington, Seattle, Washington, United States of America; University of Saarland Medical School, Germany

## Abstract

Several risk factors have been identified as potential contributors to pancreatic cancer development, including environmental and lifestyle factors, such as smoking, drinking and diet, and medical conditions such as diabetes and pancreatitis, all of which generate oxidative stress and DNA damage. Oxidative stress status can be modified by environmental factors and also by an individual's unique genetic makeup. Here we examined the contribution of environment and genetics to an individual's level of oxidative stress, DNA damage and susceptibility to pancreatic cancer in a pilot study using three groups of subjects: a newly diagnosed pancreatic cancer group, a healthy genetically-unrelated control group living with the case subject, and a healthy genetically-related control group which does not reside with the subject. Oxidative stress and DNA damage was evaluated by measuring total antioxidant capacity, direct and oxidative DNA damage by Comet assay, and malondialdehyde levels. Direct DNA damage was significantly elevated in pancreatic cancer patients (age and sex adjusted mean ± standard error: 1.00±0.05) versus both healthy unrelated and related controls (0.70±0.06, p<0.001 and 0.82±0.07, p = 0.046, respectively). Analysis of 22 selected SNPs in oxidative stress and DNA damage genes revealed that *CYP2A6* L160H was associated with pancreatic cancer. In addition, DNA damage was found to be associated with *TNFA* −308G>A and ERCC4 R415Q polymorphisms. These results suggest that measurement of DNA damage, as well as select SNPs, may provide an important screening tool to identify individuals at risk for development of pancreatic cancer.

## Introduction

Pancreatic cancer, the fourth leading cause of cancer deaths in the United States, is characterized by rapid metastasis and profound resistance to chemo- and radiotherapy. Detection late in the disease course and limited treatment options contribute to its poor prognosis [1], with median 5 year survival rates of 6% [2]. As environmental factors play a significant role in the etiology of sporadic pancreatic cancer [3], identification of gene-environment interactions that contribute to pancreatic cancer oncogenesis is essential for disease prevention. Further, development of diagnostic tests which can identify susceptible individuals or monitor disease progression may aid in prevention or guide pancreatic cancer treatment.

In addition to chronic pancreatitis and diabetes, several lifestyle risk factors have been linked to the development of pancreatic cancer, including smoking, heavy alcohol consumption, and obesity [3], [4]. A common feature of these risk factors is their ability to induce oxidative stress and DNA damage [5]. Oxidative stress is defined as an imbalance between the production of reactive oxygen species (ROS) and their elimination and repair by cellular defense mechanisms. By causing damage to lipid, protein, and DNA, ROS contribute to the pathology observed in chronic inflammatory conditions, aging, and cancer [6]–[9]. Cellular defense mechanisms exist to both repair damaged DNA and detoxify ROS. Oxidatively modified bases and single-strand DNA breaks are primarily repaired by the base excision DNA repair (BER) pathway, while bulky adducts are repaired by the nucleotide excision repair (NER) pathway [10]. Enzymatic antioxidants such as superoxide dismutase (SOD), nitric oxide synthase (NOS), catalase (CAT) and non-enzymatic antioxidants such as glutathione, vitamin C and vitamin D serve to neutralize ROS [6]. Biological markers which quantify oxidative stress include measurements of total antioxidant capacity (TAC), lipid peroxidation products, such as malondialdehyde (MDA), and DNA damage, which is commonly assessed by the Comet assay [11]. In circulating peripheral blood mononuclear cells (PBMCs), increased DNA damage has been observed in cigarette smokers [12], and in type 2 diabetic patients which correlated with hyperglycemia [13], [14]. Increased lipid peroxidation levels, concomitant with decreased TAC, were also seen in patients with type 2 diabetes and chronic pancreatitis [14], [15]. With respect to pancreatic malignancies, activation of the DNA damage response pathway has been documented in pre-cancerous pancreatic intraepithelial neoplasia [16], and dysregulation of oxidative stress-related pathways such as Nrf2/Keap1 have been observed in pancreatic cancer cell lines and human tumors [17].

An individual's oxidative stress level depends on lifestyle determinants, such as smoking, drinking and diet, and is also influenced by genetics. Several case-control studies have investigated the correlation of single nucleotide polymorphisms (SNPs) in genes related to carcinogen metabolism, oxidative stress and DNA repair, with pancreatic cancer. While SNPs in the phase I and II metabolism genes *CYP1A1*, *GSTM1*, *GSTT1* and *GSTP1* alone did not correlate with pancreatic cancer risk, a significant interaction between smoking and the *GSTT1* null genotype was reported in Caucasian pancreatic cancer subjects [18]. Investigation of the NER pathway revealed an association of SNPs in the *MMS19L* gene with pancreatic cancer risk [19]. A decreased pancreatic cancer risk was observed for carriers of *ERCC4* R415Q and *LIG3* G-39A minor alleles, while an increased risk was observed for the *ATM* D1853N allele [20], [21]. Polymorphisms in DNA repair genes have also been linked to pancreatic cancer risk in the context of exposure to smoking or individual history of diabetes [20], [22]. However, other studies failed to identify direct correlations of SNPs in metabolism and DNA repair genes with pancreatic cancer risk [23], [24]. The present pilot study examines the role of environmental factors and genetics in pancreatic carcinogenesis by evaluating biological measurements of oxidative stress, DNA damage, and specific lifestyle factors and genetic polymorphisms among groups of pancreatic cancer patients and healthy genetically related and unrelated controls.

## Materials and Methods

### Ethics Statement

This study was approved by the Indiana University Institutional Review Board. Written informed consent was obtained from participants.

### Study Population

A total of 31 patients (cases) with pathologically confirmed pancreatic cancer (Stages I–IV) and 40 healthy controls (20 genetically related and 20 unrelated) were enrolled. Cases were excluded if they had a history of other malignancies, or had already received treatment with chemotherapy or radiotherapy. Cases were matched with either genetically related controls and/or genetically unrelated controls. These distinct control groups were recruited in order to discern the contribution of environmental and genetic factors in pancreatic cancer risk. Genetically related controls were included provided they did not live with their matched case while genetically unrelated controls had to be cohabiting with the case. All enrolled participants were Caucasian, ≥18 years at time of consent, and able to understand and sign a written informed consent. Information concerning subject demographics, behavioral factors (diet, smoking, alcohol, occupational exposures), and personal and family medical history were obtained by self-report using a questionnaire at the time of enrollment. Cumulative smoking was calculated as pack-years [(packs/day)x(years smoked)]. Alcohol consumption was reported as days drinking in the past year. Body mass index (BMI, kg/m^2^) was calculated from self-reported height and weight or patient's charts. Blood samples were obtained from participants at the time of enrollment.

### Measurement of Total Antioxidant Capacity

Total antioxidant capacity (TAC) was measured in serum as described [25]. A standard curve was generated using Trolox (Sigma, St. Louis, MO) and TAC quantified from the standard curve.

### Assessment of Direct and Oxidative DNA Damage: Comet Assay

Whole blood (10 µl) was mixed with 0.5 ml RPMI 1640 containing 10% FBS, 10% DMSO, 1 mM deferoxamine, step-frozen and stored at −80°C until analysis. The Comet assay was performed as described previously [26]. Briefly, 6 µl of blood was mixed with 70 µl 1% low melting point agarose and applied onto Trevigen CometSlides®. Cells were lysed, placed in alkali buffer, and then electrophoresed. Slides were stained with ethidium bromide and 100 randomly selected nuclei/sample were evaluated (Komet 4.0; Kinetic Imaging Ltd., Liverpool, UK). Oxidative DNA damage was assessed using enzymatic digestion with formamidopyrimidine-DNA glycosylase (fpg) prior to electrophoresis. DNA damage was expressed as Comet (Olive) tail moment [(tail mean – head mean)*tail%DNA/100].

### Measurement of Malondialdehyde (MDA)

MDA was measured in serum using high performance liquid chromatography (HPLC) with UV detection as described previously [27].

### DNA Isolation and Genotyping

Genomic DNA was extracted from PBMCs in whole blood using QIAamp DNA Blood Midi kit (Qiagen). Determination of SNPs by allelic discrimination was performed using TaqMan validated probes (Applied Biosystems) on an ABI 7900HT instrument according to manufacturer's instructions. The SNPs were selected based on their previously reported associations with oxidative stress status, DNA damage, and cancer risk.

### Statistical Analysis

Generalized and linear mixed models that accounted for the correlations between patients and controls were used to compare demographic, environmental factors, and chronic pancreatitis and other inflammatory conditions between the three arms. Oxidative stress and DNA damage measures were compared between the pancreatic cancer patients and healthy unrelated or healthy related controls and correlated with environmental factors and SNPs (dominant, recessive, and gene dose models) using linear mixed models adjusting for age and sex due to group differences in these demographics. Adjusted means ± standard errors are reported from these models. Hardy Weinberg equilibrium test was conducted to check the genotype QC. Genetic risk of pancreatic cancer was assessed by comparing SNP frequencies between patients and unrelated healthy controls using Fisher exact tests (for dominant and recessive genetic models) or Mantel-Haenszel Chi-square exact tests (for gene dose models). All analyses were performed using SAS Version 9.3 (Cary, NC). P-values of <0.05 were considered statistically significant. The purpose of this pilot study was to generate hypotheses, especially genetic signals. Therefore, the type I error was not stringently controlled in multiple-comparisons.

## Results

### Study Subject Characteristics

Demographic characteristics of the case and control subjects are shown in Table 1. Cases were significantly older than the healthy related control group; however, age of the healthy unrelated control group was similar to that of the cases. Cases were more likely to be male compared to both control groups while BMI was similar between all groups. Both healthy unrelated and related controls were more likely to be never-smokers compared to pancreatic cancer cases (53% and 67% versus 38%, respectively) although the differences were marginally significant (p = 0.07). Smoking ≥20 pack years was similar in cases and healthy unrelated controls compared to healthy related controls. Alcohol consumption in the past year was not statistically different among the groups. Chronic pancreatitis and other inflammatory conditions, which included chronic pancreatitis, diabetes, and inflammatory bowel disease, were significantly increased in pancreatic cancer patients.

**Table 1:** Demographics and Risk Factors by Study Arm.

	Pancreatic Cancer Cases (n = 31)	Healthy Unrelated Control (n = 20)	Healthy Related Control (n = 20)	p-value
Age				
Mean ± SD	64.9±9.6	62.1±11.2	48.8±12.1	<.001
Range	46–84	45–87	20–69	
Sex				
Male	19 (61%)	5 (25%)	5 (25%)	.01
Female	12 (39%)	15 (75%)	15 (75%)	
BMI^a^				
Mean ± SD	25±5	28±6	27±5	.09
Range	17–37	17–42	21–38	
Smoking^a^: n (%)				
Never	11 (38%)	10 (53%)	12 (67%)	.07
Former	10 (34%)	4 (21%)	5 (28%)	
Current	8 (23%)	5 (26%)	1 (6%)	
<20 pack years	19 (66%)	13 (68%)	15 (83%)	.23
≥20 pack years	10 (34%)	6 (32%)	3 (17%)	
Alcohol, past year consumption^a^: n (%)				
≤7 days	15 (52%)	10 (52%)	8 (44%)	.72
8–30 days	5 (17%)	6 (32%)	5 (28%)	
>30 days	9 (31%)	3 (16%)	5 (28%)	
Chronic Pancreatitis^b^: n (%)				
Yes	7 (23%)	0 (0%)	1 (5%)	.03
No	23 (77%)	19 (100%)	18 (95%)	
Inflammatory Conditions^b^: n (%)				
Yes	13 (43%)	3 (16%)	3 (16%)	.02
No	17 (57%)	16 (84%)	16 (84%)	

a Missing data for 2 pancreatic cancer cases; 1 healthy unrelated control; and 2 healthy related controls;

b Missing data for 1 pancreatic cancer case; 1 healthy unrelated control; and 1 healthy related controls.

### Association of Pancreatic Cancer and Biomarkers of Oxidative Stress

An oxidative stress/damage profile was generated from blood samples of all enrolled participants (Table 2). Total antioxidant capacity (TAC), measured by the Trolox-equivalent antioxidant assay, was similar between pancreatic cancer cases and healthy unrelated controls. In contrast, cases exhibited higher TAC levels compared to healthy related controls; however, this difference was not significant. Levels of the lipid peroxidation product MDA were similar in pancreatic cancer cases compared to the healthy unrelated controls and were higher in cases versus healthy related controls, but did not achieve significance.

**Table 2:** Biomarkers of Oxidative Stress and Damage by Study Arm.^a^

	Pancreatic Cancer Cases (n = 31)	Healthy Unrelated Control (n = 20)	Healthy Related Control (n = 20)	Cancer v. Unrelated Control	Cancer v. Related Control
	Adjusted Mean ± SE	Adjusted Mean ± SE	Adjusted Mean ± SE	p-value	p-value
TAC^b^ (mM)	4.50±0.08	4.55±0.10	4.43±0.11	0.66	0.65
Direct DNA Damage^c^	1.00±0.05	0.70±0.06	0.82±0.07	<0.001	0.046
Oxidative DNA Damage	1.79±0.14	1.56±0.17	1.43±0.19	0.26	0.15
MDA^d^ (µM)	1.24±0.05	1.22±0.06	1.15±0.07	0.81	0.33

a Adjusted for age and sex;

b TAC = Total antioxidant capacity;

c DNA damage (direct and oxidative is expressed as Comet (Olive) tail moment: [(tail mean – head mean)*tail%DNA/100];

d MDA = malondialdehyde.

The alkaline Comet assay, that measures single and double DNA strand breaks, was used to assess direct DNA damage in PBMCs of cases and controls. Oxidative DNA damage was determined by a modified Comet assay which included incubation of the sample with formamidopyrimidine-DNA glycosylase (fpg) prior to electrophoresis. Fpg recognizes oxidatively modified purines and introduces additional DNA strand breaks, thus the difference between fpg-modifed and un-modified Comet measurements corresponds to oxidatively damaged DNA. As shown in Table 2, oxidative DNA damage was higher in pancreatic cancer cases versus both healthy unrelated and healthy unrelated controls; however, differences were not statistically significant. Levels of direct DNA damage were significantly elevated in cases (1.00±0.05) versus both healthy unrelated and related controls (0.70±0.06, p<0.001 and 0.82±0.07, p = 0.046, respectively).

Next, we examined potential correlations between known pancreatic cancer risk factors and oxidative stress biomarkers (Table 3). All correlations were adjusted for age and sex. Correlations between smoking and oxidative stress markers did not achieve statistical significance with the exception of direct DNA damage, where a significant negative correlation was noted (r_s_ = −.29, p = .02). No significant associations were observed between days drinking and TAC, direct, or oxidative DNA damage. No correlations between oxidative stress markers and chronic pancreatitis were observed; however, overall, inflammatory conditions were associated with elevated levels of MDA (p = 0.048).

**Table 3:** Correlation Between Oxidative Stress/Damage Markers and Environmental Factors^a^.

	n	TAC (mM)	Direct DNA Damage	Oxidative DNA Damage	MDA (µM)
		Adjusted Mean ± SE	Adjusted Mean ± SE	Adjusted Mean ± SE	Adjusted Mean ± SE
Smoking^b^		p = .64	p = .38	p = .34	p = .50
Never	33	4.48±0.08	0.90±0.05	1.52±0.15	1.21±0.05
Former	19	4.48±0.10	0.80±0.06	1.88±0.19	1.14±0.07
Current	14	4.60±0.12	0.80±0.07	1.64±0.21	1.25±0.07
Chronic Pancreatitis^c^		p = .21	p = .96	p = .75	p = .81
Yes	8	4.68±0.16	0.86±0.10	1.73±0.28	1.19±0.10
No	60	4.46±0.06	0.86±0.04	1.63±0.11	1.22±0.04
Inflammatory Conditions^c^		p = .77	p = .08	p = .25	p = .048
Yes	19	4.52±0.10	0.96±0.06	1.48±0.18	1.32±0.07
No	49	4.48±0.07	0.82±0.04	1.72±0.12	1.16±0.04
		r_s_	r_s_	r_s_	r_s_
Pack Years	66	0.18 p = .18	−0.29 p = .02	0.03 p = .86	−0.04 p = .75
Days Drinking Alcohol in past Year^b^	66	0.03 p = .82	0.18 p = .15	−0.03 p = .78	−0.17 p = .16

a Adjusted for age and sex;

b Missing data for 2 pancreatic cancer cases; 1 healthy unrelated control; and 2 healthy related controls;

c Missing data for 1 pancreatic cancer case; 1 healthy unrelated control; and 1 healthy related controls.

### Association of Pancreatic Cancer and Direct DNA Damage with SNP Expression

The genotype of 22 SNPs in 17 genes involved in oxidative stress, inflammation, DNA damage, methionine/folate metabolism and carcinogen metabolism was determined in pancreatic cancer cases and control groups (Table S1). Analysis of the contribution of individual SNPs to pancreatic cancer was determined by comparing SNP genotype in cases versus healthy unrelated controls (Table 4). The *CYP2A6* L160H major allele was associated with pancreatic cancer overall (p = 0.03), as well as exhibiting significance for dominance, recessive and gene dose effects.

**Table 4:** Significance of SNP Expression in Pancreatic Cancer Cases vs. Healthy Unrelated Control.

				p Values			
Function	Gene	Change	rs #	Overall	Dominance	Recessive	Gene Dose
***Inflammation***	TNFA	−308 G>A	1800629	0.875	1.000	1.000	1.000
	TLR4	D299G	4986790	0.077	0.514	0.411	1.000
		T399I	4986791	0.264	0.514	0.668	1.000
***Oxidative Stress***	SOD2	A16V	4880	0.573	0.456	0.755	1.000
	GSTM1	K173N	1065411	0.836	0.685	0.657	0.657
	GSTM3	V224I	7483	0.797	1.000	0.545	0.676
	NOS3	D298E	1799983	0.835	1.000	0.773	0.647
	CAT	−21 A>T	7943316	0.511	0.389	0.384	0.277
		−262 C>T	1001179	0.815	0.640	1.000	1.000
***DNA damage***	APEX1	I64V	2307486	1.000	–a	1.000	1.000
		Q51H	1048945	0.640	0.640	–a	1.000
	OGG1	S236C	1052133	0.856	0.767	0.565	0.676
	ERCC2	D312N	1799793	0.697	0.514	0.778	0.622
		D711D	1052555	0.204	0.566	0.143	0.206
		Q751K	13181	0.513	0.567	0.384	0.308
	ERCC4	R415Q	1800067	0.070	0.070	–a	0.070
	XRCC1	Q399R	25487	0.923	1.000	0.773	0.675
	ATM	D1853N	1801516	1.000	1.000	1.000	0.779
***Methionine/folate metabolism***	MTRR	H595Y	10380	1.000	1.000	1.000	1.000
	MTHFR	A222V	1801133	0.502	1.000	0.249	0.456
***Metabolism***	CYP2A6	L160H	1801272	0.029	0.029	0.055	0.016
	UGT1A7	W208R	1.2E+07	0.892	0.778	1.000	0.802

a – = no corresponding dominant (or recessive) nucleotide.

Since direct DNA damage was significantly elevated in pancreatic cancer cases compared to both control groups, the correlation between SNP genotype and this biomarker were analyzed (Tables 5 and 6). The AA variant of the *TNF* −308 G>A SNP exhibited a significant recessive effect (p = 0.003). The *ERCC4* R415Q polymorphism demonstrated significant dominant and gene dose effects (p = 0.009). For both genes, the minor allele resulted in a significant elevation in direct DNA damage (Table 6). The AA variant of the *TNFA* −308 G>A SNP was observed in only 2 subjects with a wide standard deviation in direct DNA damage levels; however a statistical difference was still achieved between DNA damage in the AA (1.44±0.19) versus the AG (0.81±0.06) and GG (0.88±0.04) alleles. While no QQ homozygous individuals were present in this study, heterozygous *ERCC4* R415Q subjects exhibited elevated DNA damage versus RR individuals (1.12±0.10 and 0.84±0.04 respectively).

**Table 5:** Correlation of SNPs with Direct DNA Damage.

SNP	Genotype			p values		
				Dominance	Recessive	Gene Dose
TNFA(−308G>A)	AA	AG	GG	0.877	0.003	0.408
TLR4(D299G)	AA	AG	GG	0.881	0.243	0.390
TLR4(T399I)	CC	CT	TT	0.881	0.105	0.240
SOD2(A16V)	AA	AG	GG	0.752	0.682	0.654
GSTM1(K173N)	CC	CG	GG	0.310	0.097	0.169
GSTM3(V224I)	CC	CT	TT	0.541	0.817	0.631
NOS3(D298E)	GG	GT	TT	0.561	0.638	0.534
CAT (−21A>T)	AA	AT	TT	0.530	0.792	0.730
CAT (−262C>T)	CC	CT	TT	0.665	0.603	0.561
APEX1(I64V)	AA	AG	GG	–a	0.234	0.234
APEX1(Q51H)	CC	CG	GG	0.414	–a	0.414
OGG1(S326C)	CC	CG	GG	0.073	0.135	0.097
ERCC2(D312N)	GG	GA	AA	0.144	0.152	0.073
ERCC2(D711D)	AA	AG	AA	0..097	0.560	0.116
ERCC2(Q751K)	GG	GT	TT	0.292	0.813	0.476
ERCC4(R415Q)	AA	AG	GG	0.009	–a	0.009
XRCC1(Q399R)	CC	CT	TT	0.629	0.305	0.331
ATM(D1853N)	AA	AG	GG	0.397	0.849	0.394
MTRR(H595Y)	CC	CT	TT	0.334	0.396	0.301
MTHFR(A222V)	GG	GA	AA	0.207	0.330	0.181
CYP2A6(L160H)	TT	TA	AA	0.765	0.857	0.906
UGT1A7(W208R)	CC	CT	TT	0.340	0.400	0.249

a – = no corresponding dominant (or recessive) nucleotide.

**Table 6:** Correlation of Genotype with Direct DNA Damage^a^.

Gene	SNP	Nucleotide	Amino Acid	n	Direct DNA Damage Adjusted Mean ± SE
TNFA	−308 G>A	AA	–	2	1.44±0.19^b^
		AG	–	19	0.81±0.06
		GG	–	50	0.88±0.04
ERCC4	R415Q	AA	QQ	0	–
		AG	RQ	8	1.12±0.10^b^
		GG	RR	63	0.84±0.04

a Adjusted for age and sex;

b Statistically different from other genotypes (p<0.05);

## Discussion

The etiology of sporadic pancreatic cancer remains largely unknown; however, epidemiologic studies have identified risk factors that contribute to the development of pancreatic carcinomas. These factors include environmental and lifestyle factors, such as smoking, drinking and diet, and inflammatory conditions such as diabetes and pancreatitis, all of which share the ability to generate oxidative stress and DNA damage. The present pilot study was designed to examine the contribution of environment and genetics to an individual's level of oxidative stress and DNA damage and the subsequent risk for development of pancreatic cancer. To accomplish this objective, we utilized three groups of subjects; a pancreatic cancer cohort, a healthy unrelated control group living with the case subject, and a healthy genetically related control group which did not reside with the case. We found evidence to suggest that both environment and genetics contribute to the oxidative stress and DNA damage observed in cases and controls in our study.

Oxidative stress and DNA damage were evaluated using four measurements: TAC, direct and oxidative DNA damage measured in circulating PBMCs, and MDA levels. These parameters have previously been investigated in human diabetic subjects and in an animal model of diabetes [13], [14], [28], [29]; however, to date this is the first report of these parameters in pancreatic cancer patients. In type 2 diabetic patients, decreased TAC and increased plasma MDA was observed, compared to subjects with normal glucose tolerance [14]. We found that both cases and unrelated controls exhibited similar mean levels of TAC, MDA, and oxidative DNA damage while there were greater, though not significant, mean differences in pancreatic cancer subjects in comparison to healthy related controls (Table 2). It is possible that the upregulation of TAC seen in the pancreatic cancer cases may be due to smoking, since the percentage of current and former smokers compared to never smokers was higher in cases versus healthy related controls (Table 1). In addition, we observed a positive correlation of TAC with pack years (Table 3). Smoking has been shown in some studies to increase the activity of antioxidant enzymes [30], [31], which would lead to increased TAC.

One of the most significant observations in this study was an increased level of direct DNA damage in the pancreatic cancer group compared to both control groups (Table 2). This observation may be related to the increased incidence of inflammatory conditions observed among cases (Table 1). Our results are consistent with the increased direct DNA damage previously reported in diabetes, which is an inflammatory condition [13], [14], [28]. Oxidative damage to bases can be determined using modified Comet assays, which use repair endonucleases to assess specific types of damage. An increase in endonuclease III-sensitive sites, indicative of oxidized pyrimidine bases, was observed in type 2 diabetes, while an increase in fpg-sensitive sites, indicative of oxidized purines, was found in some but not all studies [13], [28], [29]. In our study, while fpg-modified oxidative DNA damage was higher in pancreatic cancer patients compared to both control groups, the increase was not statistically significant. Together, our results indicate that a component of oxidative stress and DNA damage may be environmentally-related, as both cases and unrelated healthy controls exhibit similar levels of TAC, MDA and oxidative DNA damage. However, the environment alone cannot account for the difference observed in direct DNA damage, as these were significantly elevated in pancreatic cancer cases versus both control groups.

We next looked at the contribution of genetics as a modifier of pancreatic cancer risk. We focused our analysis on pathways related to known pancreatic cancer risk factors: carcinogen metabolism, inflammation, and DNA damage and repair. Cigarette smoke can generate free radicals and oxidants which can lead to increased oxidative stress while it also contains procarcinogenic compounds that can be metabolized to potent carcinogens. While correlations between smoking and oxidative stress biomarkers were not observed, an association was seen between pancreatic cancer and the *CYP2A6* L160H polymorphism. Among its diverse substrates, CYP2A6 catalyzes the metabolism of nicotine and tobacco-specific procarcinogens, such as 4-(methylnitrosamine)-a-(3-pyridyl)-1-butanone or NNK [32]. CYP2A6 expression and activity levels are highly variable in individuals, largely due to genetic polymorphisms. High enzymatic activity of CYP2A6 has been associated with lung, esophageal and colorectal cancer [32], [33]. The L160H polymorphism in CYP2A6 results in the expression of a protein with no enzymatic activity [34]. Individuals who possess the *CYP2A6* His variant would be unable to activate procarcinogens in cigarette smoke and thus would be protected against cancer development, a finding that has been shown in lung cancer [35]. In our study, we found that the majority (97%) of pancreatic cancer cases possess the enzymatically active homozygous AA *CYP2A6* allele compared to 75% and 89% of the healthy unrelated and related controls, respectively (Table S1). These results are in agreement with a recent study which found an 80% increased risk for development of pancreatic cancer in individuals who exhibited the highest quartile of CYP2A6 activity [36].

Overexpression of tumor necrosis factor alpha (TNFA), a pro-inflammatory cytokine, has been implicated in autoimmune diseases and cancers associated with an inflammatory component [37], [38]. In this study, chronic pancreatitis and other inflammatory conditions were more prevalent in the pancreatic cancer cases (Table 1). In addition, elevated MDA levels were significantly associated with inflammatory conditions, and direct DNA damage approached statistical significance in individuals with inflammatory conditions (Table 3). The A allele at position −308 in the TNFA gene promoter has been shown to correlate with elevated TNFα expression [38]. In a previous study of pancreatic cancer patients, pancreatitis was associated with −308 *TNFA* GA + AA alleles; however, no overall association with pancreatic cancer was seen [39]. In addition, the −308A allele conferred a 2-fold risk for development of type 2 diabetes [40]. In the present study, we found an association between the homozygous −308 A *TNFA* allele and direct DNA damage (Table 5 and 6); however, consistent with previous studies [39], we did not observe an association with pancreatic cancer (Table 4). These results suggest that individuals harboring the −308 A *TNFA* allele may be prone to develop chronic inflammatory conditions, which may lead to DNA damage and pancreatic cancer.

In our study, DNA damage in PBMCs was significantly elevated in pancreatic cancer patients (Table 3). Repair of damaged DNA is critical for prevention of DNA mispairing, genomic instability and DNA strand breaks. The BER pathway repairs oxidatively damaged DNA bases and modifications which do not distort the overall DNA structure, while the NER pathway repairs damage resulting from bulky adducts and those that distort the DNA helix, such as those caused by tobacco-related carcinogens. Multiple studies support a role for SNPs in both the NER and BER pathways in pancreatic cancer [18], [19], [21], [22]. ERCC4 is part of the ERCC1-ERCC4 endonuclease complex involved in the NER pathway [41], [42]. While homozygous R415Q *ERCC4* minor alleles (AA) have been associated with increased risk of breast cancer in several studies [43], [44], carriers of one or two minor alleles were found to have a decreased risk for pancreatic cancer [21]. The effect of the R415Q *ERCC4* polymorphism on enzyme activity has not been firmly established; however, modeling programs have predicted that the R415Q change would negatively impact protein function and repair capacity [21]. In our study, the RQ415 allele was associated with increased direct DNA damage. Of the 8 individuals which possessed the heterozygous alleles and displayed elevated DNA damage, 6 were pancreatic cancer cases and 2 were healthy related control subjects (Table 6), suggesting that the increased DNA damage observed in the *ERCC4* R415Q heterozygotes contributes to development of pancreatic cancer. This is in agreement with a recent study showing that heterozygous R415Q *ERCC4* was associated with benign breast disease, a known breast cancer precursor [45].

While these results show associations of DNA damage with pancreatic cancer and an association of DNA damage with selective SNPs, several limitations exist in our study. The low frequency of rare alleles for some SNPs may lead to spurious results whereby recruitment of additional subjects will be required to validate these findings. Recall bias may have led to misclassification of subjects into environmental lifestyle groups, as many of the identified pancreatic cancer risk factors (ie smoking, drinking) were self-reported. Age was significantly lower in the healthy related control group compared to pancreatic cancer subjects. Since increased DNA damage has been observed with aging [12], we have adjusted for age in our analyses. However, age differences cannot account for the observed association between direct DNA damage and pancreatic cancer, as age range of the cases and healthy unrelated controls was similar. All of our subjects were Caucasian, such that these findings may not extend to other ethnic groups. Our study investigated the involvement of polymorphisms in only a small subset of genes; thus many potential gene-gene interactions and gene-environment interactions remain to be studied.

The use of two control groups in this study enabled the investigation of the contribution of environment to pancreatic cancer in a unique way; control subjects who reside with cases are more likely to share the same lifestyle exposures, as opposed to controls that are matched to cases by questionnaire data alone. In addition, these potential exposures are likely to have been shared for a considerable amount of time which enables a more accurate assessment of the contribution of individual factors to pancreatic cancer development. Overall, results from this pilot study support a role of both genetics and environmental/lifestyle factors in the development of pancreatic cancer. We report an association of pancreatic cancer with DNA damage and associations with specific polymorphisms in genes involved in metabolism (*CYP2A6*), inflammation (*TNFA*), and DNA damage and repair (*ERCC4*). Evaluating DNA damage in circulating PBMCs, as well as select genotyping strategies may thus provide an important screening tool to identify individuals at an increased risk for developing pancreatic cancer. Due to the small sample size in this pilot study, assessment of these endpoints in additional pancreatic cancer and control subjects is needed. In addition, mechanistic studies of individual SNPs in *CYP2A6*, *TNFA* and *ERCC4* genes would be useful to assess their contribution to the development of pancreatic cancer.

## Supporting Information

Table S1 **Genotype Frequencies in Selected SNPs.**
(DOCX)
