# Supplementary material for: Contribution of Environment and Genetics to Pancreatic Cancer Susceptibility
Source: PLoS One. 2014 Mar 20;9(3):e90052. doi: 10.1371/journal.pone.0090052 (PMC3961224; doi:10.1371/journal.pone.0090052)
Supplement: Table S1 — Genotype Frequencies in Selected SNPs. (DOCX) [file pone.0090052.s001.docx]

**Table S1: Genotype Frequencies in Selected SNPs**

| **rs #** | **Gene** | **SNP** | **Nucleotide Sequence** | **Pancreatic Cancer Cases**  **N=31**  **n (%)** | **Healthy Unrelated Control**  **N = 20**  **n (%)** | **Healthy Related Control**  **N=20**  **n (%)** |
| --- | --- | --- | --- | --- | --- | --- |
| 1800629 | TNFA | -308 G>A | AA | 1 (3) | 1 (5) | 0 (0) |
|  |  |  | AG | 8 (26) | 4 (20) | 7 (35) |
|  |  |  | GG | 22 (71) | 15 (75) | 13 (65) |
| 4986790 | TLR4 | D299G | AA | 28 (90) | 16 (80) | 16 (80) |
|  |  |  | AG | 1 (3) | 4 (20) | 4 (20) |
|  |  |  | GG | 2 (6) | 0 (0) | 0 (0) |
| 4986791 | TLR4 | T399I | CC | 28 (90) | 17 (85) | 16 (80) |
|  |  |  | CT | 1 (3) | 3 (15) | 4 (20) |
|  |  |  | TT | 2 (6) | 0 (0) | 0 (0) |
| 4880 | SOD2 | A16V | AA | 10 (32) | 5 (25) | 5 (25) |
|  |  |  | AG | 15 (48) | 13 (65) | 9 (45) |
|  |  |  | GG | 6 (19) | 2 (10) | 6 (30) |
| 1065411 | GSTM1 | K173N | CC | 5 (16) | 4 (20) | 3 (15) |
|  |  |  | CG | 1 (3) | 0 (0) | 0 (0) |
|  |  |  | GG | 9 (29) | 4 (20) | 7 (35) |
|  |  |  | Null | 16 (52) | 12 (60) | 10 (50) |
| 7483 | GSTM3 | V224I | CC | 9 (29) | 8 (40) | 9 (45) |
|  |  |  | CT | 17 (55) | 9 (45) | 8 (40) |
|  |  |  | TT | 5 (16) | 3 (15) | 3 (15) |
| 1799983 | NOS3 | D298E | GG | 12 (39) | 9 (45) | 6 (30) |
|  |  |  | GT | 16 (52) | 10 (50) | 10 (50) |
|  |  |  | TT | 3 (10) | 1 (5) | 4 (20) |
| 7943316 | CAT | -21 A>T | AA | 5 (16) | 1 (5) | 3 (15) |
|  |  |  | AT | 16 (52) | 10 (50) | 12 (60) |
|  |  |  | TT | 10 (32) | 9 (45) | 5 (25) |
| 1001179 | CAT | -262 C>T | CC | 21 (68) | 14 (70) | 14 (70) |
|  |  |  | CT | 8 (26) | 4 (20) | 5 (25) |
|  |  |  | TT | 2 (6) | 2 (10) | 1 (5) |
| 2307486 | APEX1 | I64V | AA | 30 (97) | 20 (100) | 20 (100) |
|  |  |  | AG | 1 (3) | 0 (0) | 0 (0) |
|  |  |  | GG | 0 (0) | 0 (0) | 0 (0) |
| 1048945 | APEX1 | Q51H | CC | 0 (0) | 0 (0) | 0 (0) |
|  |  |  | CG | 2 (6) | 2 (10) | 0 (0) |
|  |  |  | GG | 29 (94) | 18 (90) | 20 (100) |
| 1052133 | OGG1 | S236C | CC | 19 (61) | 14 (70) | 14 (70) |
|  |  |  | CG | 1 (3) | 0 (0) | 0 (0) |
|  |  |  | GG | 11 (35) | 6 (30) | 6 (30) |
| 1799793 | ERCC2 | D312N | GG | 17 (55) | 12 (60) | 10 (50) |
|  |  |  | GA | 12 (39) | 8 (40) | 8 (40) |
|  |  |  | AA | 2 (6) | 0 (0) | 2 (10) |
| 1052555 | ERCC2 | D711D | AA | 5 (16) | 0 (0) | 2 (10) |
|  |  |  | AG | 9 (29) | 7 (35) | 7 (35) |
|  |  |  | GG | 17 (55) | 13 (65) | 11 (55) |
| 13181 | ERCC2 | Q751K | GG | 5 (16) | 1 (5) | 2 (10) |
|  |  |  | GT | 11 (35) | 7 (35) | 9 (45) |
|  |  |  | TT | 15 (48) | 12 (60) | 9 (45) |
| 1800067 | ERCC4 | R415Q | AA | 0 (0) | 0 (0) | 0 (0) |
|  |  |  | AG | 6 (19) | 0 (0) | 2 (10) |
|  |  |  | GG | 25 (81) | 20 (100) | 18 (90) |
| 25487 | XRCC1 | Q399R | CC | 12 (39) | 9 (45) | 6 (30) |
|  |  |  | CT | 15 (48) | 9 (45) | 9 (45) |
|  |  |  | TT | 4 (13) | 2 (10) | 5 (25) |
| 1801516 | ATM | D1853N | AA | 1 (3) | 0 (0) | 0 (0) |
|  |  |  | AG | 8 (26) | 5 (25) | 7 (35) |
|  |  |  | GG | 22 (71) | 15 (75) | 13 (65) |
| 10380 | MTRR | H595Y | CC | 25 (81) | 16 (80) | 15 (75) |
|  |  |  | CT | 5 (16) | 4 (20) | 5 (25) |
|  |  |  | TT | 1 (3) | 0 (0) | 0 (0) |
| 1801133 | MTHFR | A222V | GG | 10 (32) | 10 (50) | 7 (35) |
|  |  |  | GA | 14 (45) | 6 (30) | 10 (50) |
|  |  |  | AA | 7 (23) | 4 (20) | 3 (15) |
| 1801272 | CYP2A6 | L160H | TT | 0 (0) | 3 (15) | 0 (0) |
|  |  |  | TA | 1 (3) | 2 (10) | 2 (11) |
|  |  |  | AA | 30 (97) | 15 (75) | 17 (89) |
| 11692021 | UGT1A7 | W208R | CC | 1 (3) | 1 (5) | 3 (15) |
|  |  |  | CT | 16 (52) | 11 (55) | 6 (30) |
|  |  |  | TT | 14 (45) | 8 (40) | 11 (55) |
